# Serum Extracellular Vesicle–Derived miR-124-3p as a Diagnostic and Predictive Marker for Early-Stage Acute Ischemic Stroke

**DOI:** 10.3389/fmolb.2021.685088

**Published:** 2021-07-01

**Authors:** Zheng Qi, Yingying Zhao, Yu Su, Bin Cao, Jian-Jun Yang, Qinghe Xing

**Affiliations:** ^1^ Department of Anesthesiology, Pain and Perioperative Medicine, The First Affiliated Hospital of Zhengzhou University, Zhengzhou, China; ^2^ Institutes of Biomedical Sciences, Fudan University, Shanghai, China

**Keywords:** acute ischemic stroke, extracellular vesicle, miRNAs, inflammation, signaling pathway

## Abstract

**Background:** A delay in the diagnosis of acute ischemic stroke (AIS) reduces the eligibility and outcome of patients for thrombolytic therapy. Therefore, early diagnosis and treatment of AIS are crucial. The present study evaluated the sensitivity and accuracy of serum extracellular vesicle (EV)-derived *miR-124-3p* in the diagnosis and prediction of AIS.

**Methods:** An miRNA expression profile was downloaded from Gene Expression Omnibus (GEO) database and analyzed by R software. EVs were harvested from the serum of AIS patients using a total exosome isolation kit and characterized by Western blotting, a transmission electron microscope, and the nanoparticle tracking analysis. BV2 microglia were pre-stimulated with lipopolysaccharide (LPS), followed by *miR-124-3p* treatment for 24 h and subsequent analysis of viability, apoptosis, and migration (scratch assay), and Western blotting. The relative expression of the selected genes was assessed by qRT-PCR. The phosphorylation of Erk1/2, PI3K/Akt, and p38MAPK in BV2 microglia cells was evaluated by Western blotting, while the luciferase reporter gene assay detected the correlation between key genes involved in the pro-inflammatory signaling pathways and *miR-124-3p*.

**Results:** *hsa-miR-124-3p* was downregulated in AIS serum compared to the non-AIS serum (*p* < 0.05), and the gene expression of *has-miR-124-3p* in EVs was negatively correlated with serum pro-inflammatory cytokines and the NIHSS (*p* < 0.05). In addition, *miR-124-3p* promoted the viability and inhibited the apoptosis of LPS-induced BV2 microglia. Furthermore, *miR-124-3p* reduced the phosphorylation of Erk1/2, PI3K/Akt, and p38MAPK, and promoted the migration in LPS-induced BV2 microglia (*p* < 0.05).

**Conclusion:** Serum EV-derived *miR-124-3p* serves as a diagnostic and predictive marker for early-stage AIS.

## Introduction

Acute ischemic stroke (AIS) is one of the leading causes of death and adult disability worldwide (Bonita et al., 2004). It is a dynamic process, inducing acute brain damage and cell death (Urra et al., 2014). A delay in the diagnosis of AIS reduces the eligibility and outcome of patients for thrombolytic therapy (Hwong et al., 2016). Therefore, early diagnosis and treatment of AIS are crucial. Presently, computed tomography (CT) and magnetic resonance imaging (MRI) are considered the standard methods to diagnose AIS (Yan et al., 2017). However, the sensitivity of CT diagnosis of AIS is only 26% in the early stage (Kalafut et al., 2000). MRI presents a sensitive diagnosis of AIS, but is time-consuming and expensive, and is not available in all emergency departments (Korolev et al., 2016). Thus, there is an urgent need to explore an early alternative with a precise diagnosis of AIS. A large number of studies have been focused on investigating the role of specific markers in the diagnosis and outcome prediction in AIS (Dassan et al., 2009; Vijayan and Reddy, 2016; Zuo et al., 2020). However, due to insufficient understanding of the sensitivity and specificity of AIS standard markers, identification of these markers is challenging.

MicroRNAs (miRNAs) are a group of noncoding RNAs with ∼22 nucleotides, which regulate the biological processes posttranscriptionally by modulating the expression of target mRNAs in many diseases (Cardo et al., 2013; Leidinger et al., 2013; Hayes et al., 2014; Gurha, 2016). Reportedly, the microRNA expression profile is altered in AIS blood samples, suggesting that miRNAs are involved in the progress of AIS (Wu et al., 2012; Bhalala et al., 2013). Furthermore, several miRNAs have been identified as novel therapeutic targets for AIS (He et al., 2019; Yan et al., 2019). However, none of these have been applied in clinical practice (Harpaz et al., 2017).

Extracellular vesicles (EVs) are a group of membrane-encapsulated vesicles released by cells, biofluids, and tissues into the extracellular space, containing miRNAs, DNA, mRNAs, and proteins (Ariston Gabriel et al., 2020). EVs are involved in cell-to-cell communication despite the blood–brain barrier (BBB) and the blood–cerebrospinal fluid barrier (Skog et al., 2008; Zagrean et al., 2018). A large number of studies have described the role of EVs in stroke diagnosis, pathogenesis, and as a treatment target in the future (Deng et al., 2017; Hong et al., 2019), while a few studies demonstrated that the amount of some cell-specific EVs was increased in stroke patients compared to the controls (Jung et al., 2009; Świtońska et al., 2015; He et al., 2017).

Several EV-derived miRNAs have been altered in AIS patients. *miR-21-5p* and *miR-30a-5p* were highly expressed in AIS but decreased in the subacute phase (Wang et al., 2018). This finding indicated that the expression of EV-derived miRNAs depended on the time point of sample collection. These findings have raised the idea that EV-derived miRNAs from the serum of AIS patients may serve as analytes in the liquid biopsies for real-time monitoring of AIS severity and outcome.

In the present study, we found that the expression of *hsa-miR-124-3p* significantly increased in EVs of AIS patients, which was negatively correlated with the pro-inflammatory cytokines and the National Institutes of Health Stroke Scale (NIHSS). Additionally, we observed that *hsa-miR-124-3p* was involved in inhibiting the pro-inflammatory signaling pathways. Thus, this study might provide a novel therapeutic target and a diagnostic marker for AIS.

## Materials and Methods

### Public Data and Clinical Sample Collection

Microarray dataset (GSE95204) was obtained from the Gene Expression Omnibus (GEO) (http://www.ncbi.nlm.nih.gov/geo/). The use of human material was approved by the local Ethics Committee (reference number: 2020-KY-262, The First Affiliated Hospital of Zhengzhou University), and written consent was obtained from all patients. AIS was diagnosed by neuroimaging (CT or MRI) and stroke. Patients treated with intravenous recombinant tissue-type plasminogen activator (rt-PA) within 6 h after symptom onset were enrolled in this study. Experienced neurologists determined the neurological deficits based on the NIHSS within 6 h after the AIS onset and after rt-PA treatment. The serum samples were obtained from 10 patients at 2 h (AIS-2H, mean age: 72.5 ± 7.2 years, and range: 57–82 years), 4 h (AIS-4H, mean age: 73.0 ± 6.8 years, and range: 53–83 years), and 6 h (AIS-6H, mean age: 75.6 ± 7.2 years, and range: 56–84 years) after the onset of AIS. Control serum samples were obtained from 10 patients without AIS (mean age: 65.2 ± 8.0 years and range: 50–84 years). The fresh blood samples were collected in the vacutainer tubes, and serum was obtained by clotting at room temperature for 1 h and centrifuged at 1,500 × *g* for 10 min at 4°C (Liu et al., 2018). Mouse microglia cell line BV2 was purchased from Procell Life Science & Technology Co., Ltd. (Wuhan, China), cultured in DMEM medium (#D8437, Sigma, United Kingdom), supplemented with 10% fetal bovine serum (FBS; Sigma-Aldrich GmbH, Germany) and 1% penicillin–streptomycin at 37°C under 5% CO_2_ atmosphere.

### Analysis of Differential Gene Expression

R package and Limma package (http://bioconductor.org/packages/release/bioc/html/limma.html) were used to normalize and screen the differentially expressed miRNAs. The differential expression genes (DEGs) with a fold-change value >1 or <−1 and a *p*-value < 0.05 were considered to be significantly expressed.

### MicroRNA Target Gene Prediction

miRNA target prediction genes were predicted using TargetScan (http://www.targetscan.org/), miRDB (http://mirdb.org/), and miRTarBase (http://mirtarbase.cuhk.edu.cn/), and selected if they were recorded in at least two prediction databases (Yu et al., 2012).

### Gene Ontology and Kyoto Encyclopedia of Genes and Genomes Pathway Enrichment

Gene ontology (GO) annotation and Kyoto Encyclopedia of Genes and Genomes (KEGG) pathway enrichment analysis were enriched in miRNA-predicted target genes analyzed using the clusterProfiler (version 3.11) of R package.

### Human Serum–Derived Extracellular Vesicle Isolation and Characterization

EVs were isolated from 0.5 ml serum using a total exosome isolation kit (Thermo Fisher Scientific, Waltham, MA, United States) according to the manufacturer’s instructions. EVs were visualized by transmission electron microscopy (TEM), and their concentration was determined using the nanoparticle tracking analysis (NTA; NS300, Malvern Instruments, Malvern, United Kingdom). The classical surface markers (CD9, CD63, and CD81) of EVs were verified by Western blotting.

### RNA Extraction and Quantitative Real-Time Polymerase Chain Reaction Analysis

The total RNA of serum was isolated using the Absolutely RNA Miniprep Kit (Agilent Technologies, Santa Clara, CA, United States), according to the manufacturer’s instructions, and then cDNA was synthesized using a cDNA synthesis kit (Agilent Technologies). The RNA concentration and quality were determined using a NanoDrop^™^ (ND-1000 spectrophotometer, Thermo Fisher Scientific). The relative mRNA levels were assessed using Brilliant III Ultra-Fast SYBR^®^ Green QPCR Master Mix (Agilent Technologies) on an MX3005P QPCR System (Agilent Technologies). All genes were analyzed relatively, calibrated to the expression of control groups, and normalized to those of *GAPDH* and *18S*. All the qPCR experiments in this study were performed in duplicates using 20–50 ng cDNA.

For *hsa-miR-124-3p* expression analysis, the total RNA of cells was isolated using the miRNeasy Mini Kit (Qiagen, United States) according to the manufacturer’s instructions. *hsa-miR-124-3p* and *U6* (reference gene) were detected by TaqMan MicroRNA Assays (Applied Biosystems, Forest City, CA, United States). The sequences of all primers used for quantitative real-time polymerase chain reaction (qRT-PCR) in this study were as follows: *CXCL2*: 5′-GCC​AAG​GGT​TGA​CTT​CAA GA-3′ (forward), 5′-CTT​CAG​GGT​CAA​GGC​AAA​CT-3′ (reverse); IL1β: 5′-TGC​CAC​CTT​TTG​ACA​GTG​ATG​A-3′ (forward), 5′-TGT​GCT​GCT​GCG​AGA​TTT​GA-3′ (reverse); *IL6*: 5′-CCG​GAG​AGG​AGA​CTT​CAC​AG-3′ (forward), 5′-CAG AAT​TGC​CAT​TGC​ACA​AC-3′ (reverse); *TNF*-*α*: 5′-CTC​TGT​GAA​GGG​AAT​GGG​TG-3′ (forward), 5′-GGG​CTC​TGA​GGA​GTA​GAC​GAT​AAA​G-3′ (reverse); *GRB2*: 5′-ATT​CCT​GCG​GGA​CAT​AGA​ACA-3′ (forward) and 5′-GGT​GAC​ATA​ATT​GCG​GGG​AAA​C-3′ (reverse); *AKT3:* 5′-TGT​GGA​TTT​ACC​TTA​TCC​CCT​CA-3′ (forward) and 5′-GTT​TGG​CTT​TGG​TCG​TTC​TGT-3′ (reverse); *GAPDH*: 5′-GTG​AAG​GTC​GGT​GTG​AAC​G-3′ (forward), 5′-AATCTCCACTTTGCCACTGC-3′(reverse); *18S*: 5′-AAA​CGG​CTA​CCA​CAT​CCA​AG-3′ (forward); 5′-CCT​CCA​ATG​GAT​CCT​CGT​TA-3′ (reverse).

### Protein Extraction and Western Blotting Analysis

Serum proteins were extracted using a serum protein extraction kit (BestBio, Shanghai, China) containing phosphatase. BV2 cells were lysed in RIPA buffer (Thermo Scientific) containing phosphatase (Roche, Germany) and proteinase inhibitors. The cellular protein and EVs were quantified using a BCA Protein Kit Assay (Thermo Fisher Scientific). An equivalent amount (10 μg) of cell lysates, serum protein, or EVs was resolved by 12% sodium dodecyl sulphate–polyacrylamide gel electrophoresis (SDS-PAGE). The proteins were transferred to a 0.22-µm polyvinylidene fluoride (PVDF) membrane and blocked with 5% BSA for 1 h at room temperature. Subsequently, the membranes were incubated with primary antibodies, CD9 (ThermoFisher 10626D) 1:1,000, CD81 (ThermoFisher 10630D) 1:1,000, CD 63 (ThermoFisher 10628D) 1:500, Phospho-P38 (Cell Signaling 4,511) 1:1,000, P38 (Cell Signaling 9,212) 1:500, Phospho-Erk1/2 (Cell Signaling 4,370) 1:1,000, Erk1/2 (Cell Signaling 4,695) 1:500, Phospho-Akt (Cell Signaling 4,060) 1:1,000, and Akt (Cell Signaling 4,691) at 4°C overnight, followed by goat anti-rabbit IgG secondary antibodies (Jackson Immuno Research) for 1 h at room temperature. Protein bands were visualized using ECL detection reagents (Thermo Scientific). Image J (1.8.0, the National Institutes of Health, Bethesda, MD, United States) software was used to quantify the density of bands of phosphorylated proteins and normalize that to the total amount of proteins.

### Lipopolysaccharide Stimulation of BV2

BV2 microglial cells (2 × 10^5^/well) were cultured in 6-well plates with DMEM medium (supplemented with 10% FBS and 1% penicillin–streptomycin), stimulated with lipopolysaccharide (LPS, 50 ng/ml) (#L2630, Sigma-Aldrich) for 12 h at 37°C, 5% CO_2_, and 95% relative humidity, before the follow-up treatment and assays.

### Live-Cell Staining Assay

Calcein AM (Sigma) was utilized for simultaneous fluorescence detection. An equivalent of 2 × 10^4^ BV2 microglial cells were seeded in 24-well plates for 24 h, and then calcein AM (3 μM) was added to the culture medium. The live (green) cells were determined using a fluorescence microscope and Image J software (the National Institutes of Health, United States) for cell counting.

### *hsa-miR-124-3p* Overexpression in BV2 Microglia Cells

In order to investigate the effect of *hsa-miR-124-3p* on LPS-induced BV2 microglial cells, *hsa-miR-124-3p* mimic was transfected to overexpress *hsa-miR-124-3p* in LPS-induced BV2 microglia cells. *miR-124-3p* mimics and mimic negative control (miR-NC) were purchased from GenePharma (Shanghai, China), and the sequences were as follows: *hsa-miR-124-3p* mimic: 5′-UAA​GGC​ACG​CGG​UGA​AUG​CCA​A-3′. BV2 microglial cells (2 × 10^5^) were cultured in 6-well plates with DMEM medium (supplemented with 10% FBS and 1% penicillin–streptomycin), stimulated with LPS (50 ng/ml) for 12 h, and *hsa-miR-124-3p* mimic was transfected at a negative control using Lipofectamine® RNAiMAX Reagent (#13778075, Invitrogen, United States) at a final concentration of 100 nM. Viability assay, Caspase-3/7 assay, live assay, and migration were performed 24 h after transfection.

### Cell Viability Assay

Cell Counting Kit-8 (CCK-8) (Dojindo, Kumamoto, Japan) was utilized to measure the cell viability according to the manufacturer’s protocol. An equivalent of 5 × 10^3^ BV2 microglial cells (cultured in DMEM medium, supplemented with 10% FBS and 1% penicillin–streptomycin) were seeded into 96-well plates. After 24 h, the medium was exchanged for those containing appropriate treatment agents (LPS, *miR-124-3p* mimic, and mimic-NC). After an additional 24 h, cells were incubated with 100 µl of the fresh medium containing 10% CCK-8 reagent for 1 h at 37°C. Then the absorbance was measured at 450 nm on a Tecan ELISA reader (Mannedorf, Switzerland).

### Apoptosis Determination

To determine apoptosis of BV2 microglia cells with different treatments, caspase-3 and caspase-7 activity was evaluated using a Caspase-3/7 Assay Kit (Promega Corporation, Madison, WI, United States) according to the manufacturer’s instructions. After different treatments in a 96-well plate, a nonfluorescent caspase substrate, added to the expansion medium, was cleaved into fluorescent molecules with an emission maximum at 521 nm and evaluated on a Tecan ELISA reader (Mannedorf, Switzerland).

### Migration (Wound Healing) Assay

An equivalent of 2 × 10^5^ BV2 microglial cells (control, *hsa-miR-124-3p-mimic* transfected, and negative control) were cultured in 12-well plates. At 100% confluency, the cells were scraped away with a 100-μl pipette tip and washed twice with phosphate-buffered saline (PBS), and after changing the medium with 1% FBS, LPS (50 ng/ml) was added. Images were captured under a microscope at 0, 24, and 48 h post-wounding, and closure of the wound gap was determined using Image J (1.8.0, the National Institutes of Health).

### Luciferase Reporter Gene Assay

*GRB2* and *AKT3* as direct target genes of *miR-124-3p* were screened out based on the TargetScan database. The luciferase reporter gene assay was performed with the Dual-Luciferase Reporter Assay System (Promega). The full length of the 3′-untranslated region (UTR) of the wild-type (WT) *GRB2*/*AKT3* gene was cloned into the vector pMIR-REPORT*™* Luciferase (Promega), and the mutant-type (MUT) vectors were constructed by site-directed mutation of the binding site between *miR-124-3p* and *GRB2*/*AKT3*. The phRL-TK vector (Promega) expressing the Renilla luciferase was used to normalize the transfection efficiency. An equivalent of 1 × 10^4^ BV2 microglia were seeded in 96-well plates. At 70–80%, the reporter gene vectors (WT and MUT of *GRB2*/*AKT3*) were co-transfected with *miR-124-3p*-mimic/mimic-NC into the BV2 microglia to determine the luciferase activity, according to the manufacturer’s instructions.

### Statistical Analysis

All data are expressed as the mean ± standard deviation (SD). Statistical analysis was performed using Prism 8.02 software (GraphPad Software, United States). The comparisons between two groups were analyzed by independent two-tailed Student’s t-tests, and comparisons between more than two groups were analyzed by one-way analysis of variance (ANOVA) with the Newman–Keuls multiple comparison test. Each assay was performed in duplicate and repeated in at least three independent experiments. *p* < 0.05 was regarded as statistically significant.

## Results

### *hsa-miR-124-3p* Was Downregulated in Acute Ischemic Stroke Serum Compared to Non-Acute Ischemic Stroke Serum

**FIGURE 1:**
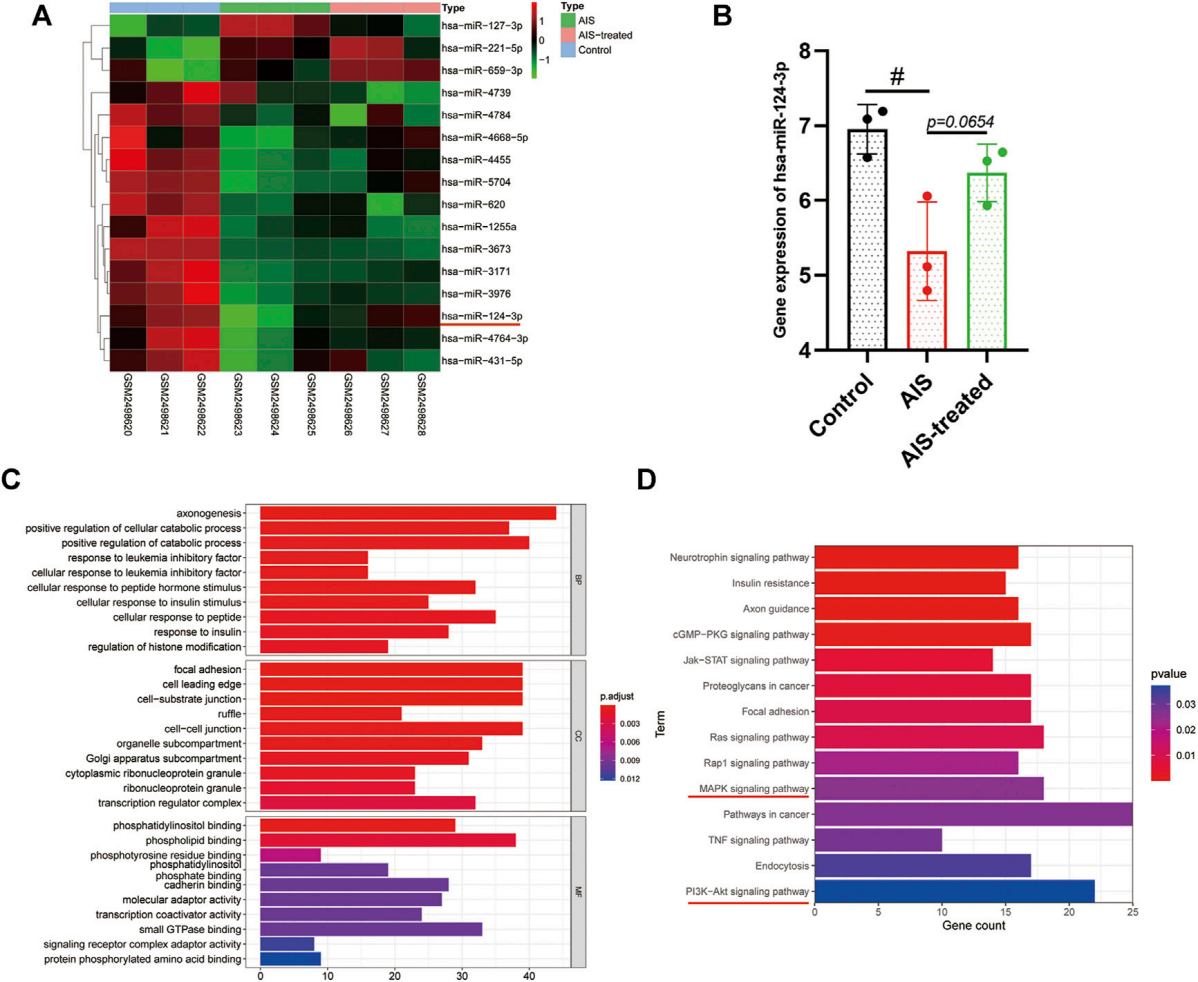
Bioinformatic analysis and the gene expression of *hsa-miR-124-3p* in AIS and AIS-treated blood samples. **(A)** Heat map of differentially expressed miRNAs in control (none-AIS), AIS, and AIS-treated samples. **(B)** Gene expression analysis of *hsa-miR-124-3p* in control (none-AIS), AIS, and AIS-treated samples. ^#^ *p* < 0.05; one-way ANOVA with the Newman–Keuls multiple comparison test. **(C,D)** GO function and KEGG pathway enrichment of targeted genes of *hsa-miR-124-3p*.

The results of miRNA (miR) microarray dataset analysis suggested that the expression of *hsa-miR-124-3p* was significantly lower in the serum of AIS patients than that of *hsa-miR-124-3p* in the serum of non-AIS (control) patients (*p* < 0.05) (Figures 1A,B). The target genes of *hsa-miR-124-3p* were identified using the public database, GO and KEGG pathway enrichment, which showed that *hsa-miR-124-3p* is involved in the regulation of pro-inflammatory signaling pathways, including PI3K/AKT and MAPK (Figures 1C,D). These data suggested that reducing the expression of *hsa-miR-124-3p* plays a critical role in the pathophysiology of AIS *via* regulating the pro-inflammatory signaling pathways.

### Isolation and Characterization of Extracellular Vesicles Derived From Serum

**FIGURE 2:**
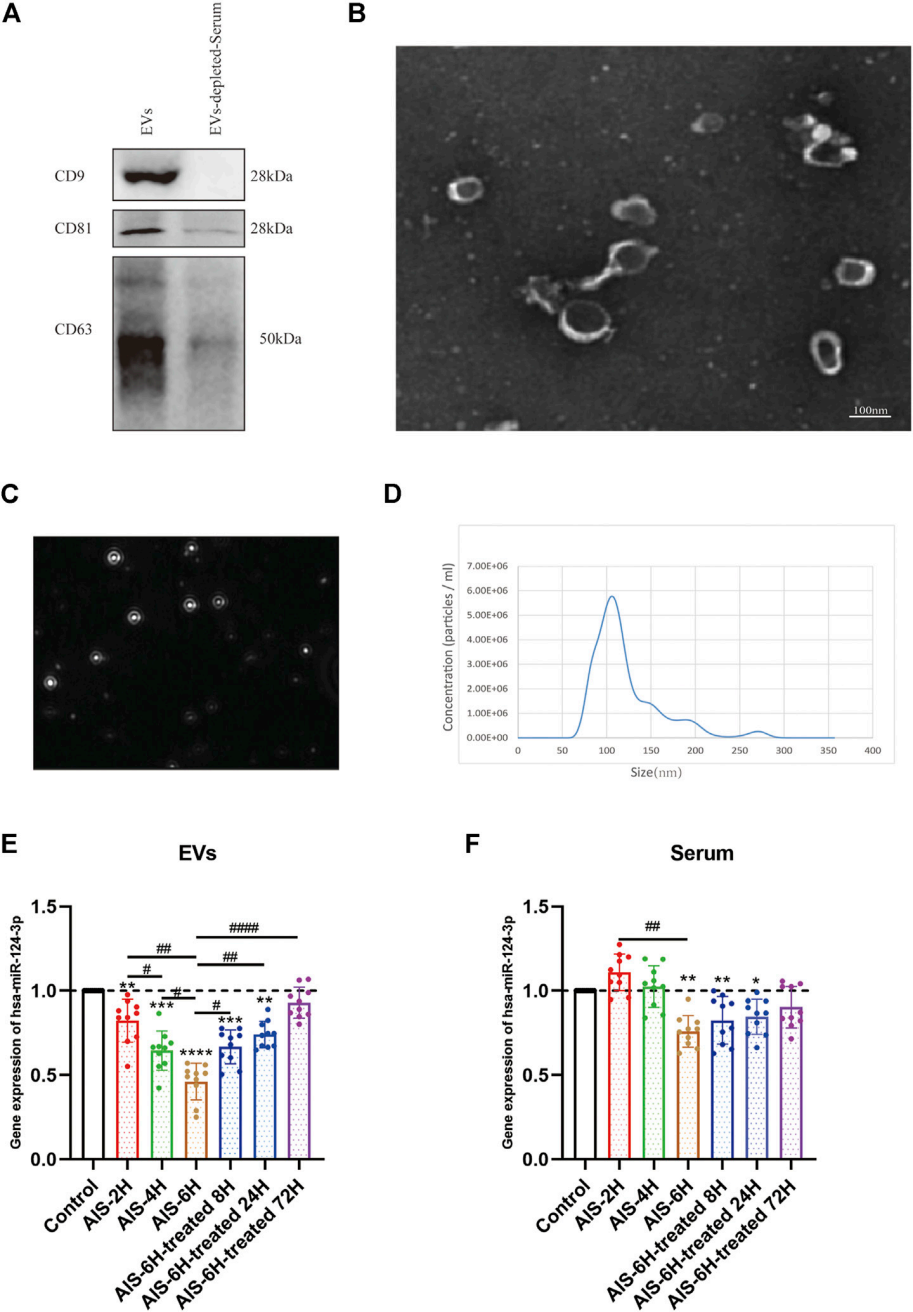
Characterization of control EVs and gene analysis of *hsa-124-3p* in serum-derived EVs and serum. **(A)** Representative image of Western blotting showed bands of standard surface markers (*CD9*, *CD63*, and *CD81*) of EVs and EV-depleted serum. **(B)** Morphology of EVs was monitored by TEM; scale bar: 100 nm. **(C,D)** Particle size distribution of EVs was determined by NTA. **(E,F)** Gene expression of *hsa-miR-124-3p* in EVs and serum of different samples at different time points. Compared to the control group: *p < 0.05; **p < 0.01; ***p < 0.001; ****p < 0.0001, ^#^ Difference between groups: ^#^ *p* < 0.05; ^##^ *p* < 0.01; ^####^ *p* < 0.0001; one-way ANOVA with the Newman–Keuls multiple comparison test.

In order to validate EVs, Western blotting was performed to verify the classical biomarkers (CD9, CD63, and CD81) of EVs (Figure 2A). The morphology of EVs was monitored by TEM (Figure 2B), and the particle size distribution was tested by NTA (Figures 2C,D), which indicated that the average diameter of EVs is approximately 110 nm.

### hsa-miR-124-3p Was Significantly Downregulated in Acute Ischemic Stroke Serum-Released Extracellular Vesicles

To investigate the correlation between AIS and *hsa-miR-124-3p*, we used qPCR to determine the expression changes of *hsa-miR-124-3p* in different samples at different time points. The AIS-6H group (6 h after the onset of AIS) was selected to verify the expression of *hsa-miR-124-3p* in EVs and serum after treatment. The results showed that *hsa-miR-124-3p* was significantly downregulated in EVs after the onset of AIS (*p* < 0.05), 4 h earlier than that in serum (Figures 2E,F). Furthermore, rt-PA treatment reversed the decreased expression of *hsa-miR-124-3p* in EVs derived from AIS serum (*p* < 0.05); however, there was no significant difference in the serum of treated groups (Figures 2E,F). These results indicated that *hsa-miR-124-3p* in EVs was more sensitive to AIS than serum.

### Gene Expression of hsa-miR-124-3p in Extracellular Vesicles Was Negatively Correlated With Pro-Inflammatory Cytokines in Serum and the National Institutes of Health Stroke Scale

**FIGURE 3:**
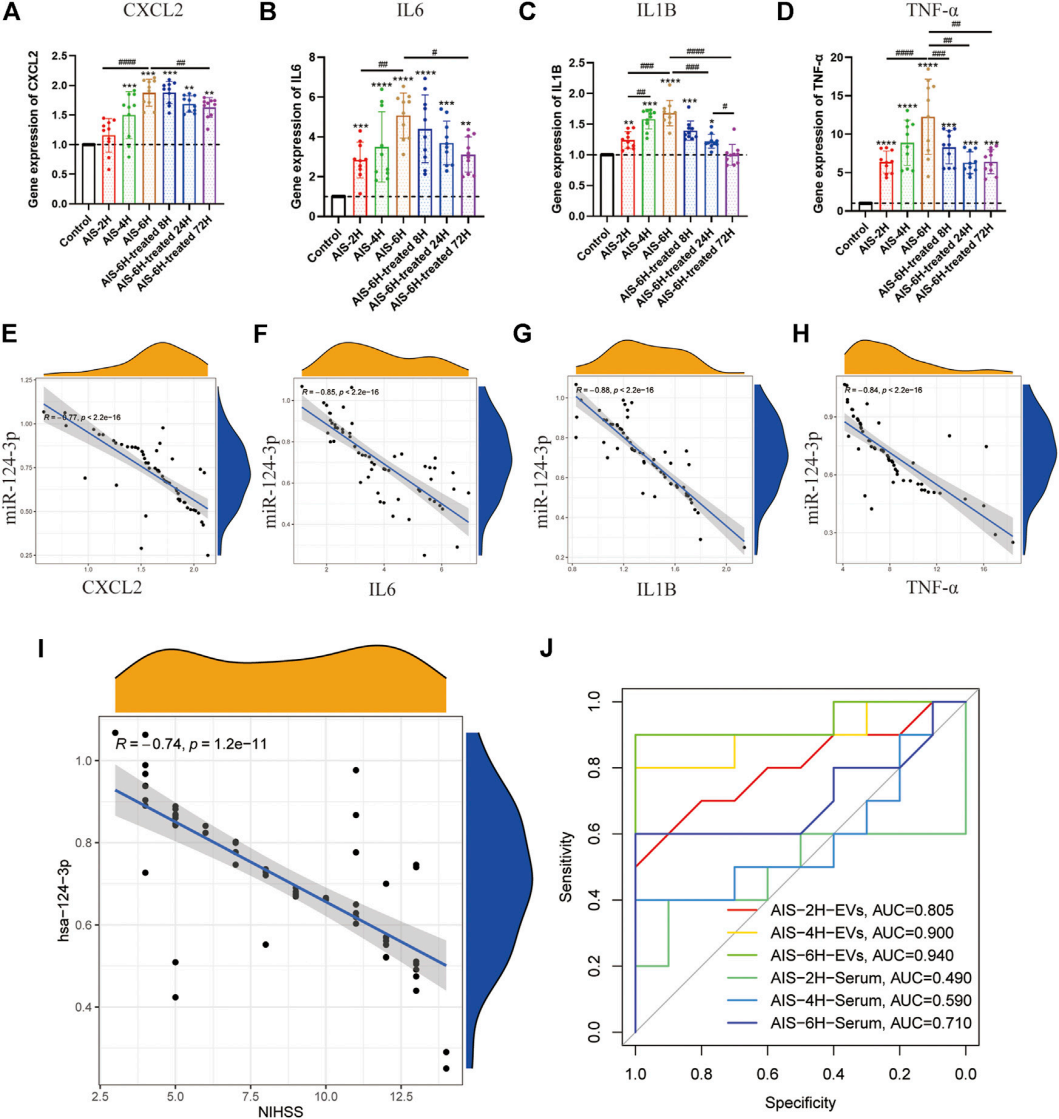
Gene expression of *hsa-miR-124-3p* in EVs negatively correlated with inflammatory factors in serum and clinical symptoms. **(A–D)** Gene expression of inflammatory factor (*CXCL2*, *IL6*, *IL1β*, and *TNF-α*) analysis in different groups at different time points. Difference to the control group: *p < 0.05; **p < 0.01; ***p < 0.001; ****p < 0.0001, ^#^ Difference between groups: ^#^ *p* < 0.05; ^##^ *p* < 0.01; ^###^ *p* < 0.001; ^####^ *p* < 0.0001; and one-way ANOVA with the Newman–Keuls multiple comparison test. **(E–H)** Correlation analysis of the gene expression between *hsa-miR-124-3p* in EVs and inflammatory factors. *R*: Pearson’s correlation coefficient, correlation coefficient |*R*| > 0.5, *p* < 0.05 was considered statistically significant. **(I)** Correlation analysis of the gene expression of *hsa-miR-124-3p* and the NIHSS. *R*: Pearson’s correlation coefficient, correlation coefficient |*R*| > 0.5; *p* < 0.05 was considered statistically significant. (**J)** ROC curve of *hsa-miR-124-3p* expression in different groups for predicting the diagnosis of AIS.

To investigate the inflammation induced by AIS, the gene expression of pro-inflammatory cytokines (*CXCL2*, *IL6*, *IL1β*, and *TNF-α*) in the serum was determined using qPCR. As shown in Figures 3A–D, as the onset time of AIS increased, the expression of pro-inflammatory cytokines (*CXCL2*, *IL6*, *IL1β*, and *TNF-α*) increased (*p* < 0.05). Additionally, the expression of all the pro-inflammatory cytokines was decreased after rt-PA treatment (*p* < 0.05), suggesting that inflammation plays a critical role in the progress of AIS.

In the present study, AIS severity and outcome were determined by the NIHSS score (Kwah and Diong, 2014). Pearson’s correlation coefficient analysis showed that hsa-miR-124-3p was negatively correlated to pro-inflammatory cytokines and the NIHSS (*p* < 0.05) (Figures 3E–I). The receiver-operating characteristic (ROC) curve of *hsa-miR-124-3p* was used to analyze the diagnostic capability of EVs and serum. Compared to the serum, EVs presented a better diagnostic capability for AIS (Figure 3J). These results demonstrated that the *hsa-miR-124-3p* in EVs derived from AIS serum might be a potential marker in the diagnosis and prognosis of AIS.

### Acute Ischemic Stroke Promoted the Phosphorylation of p38MAPK, Erk1/2, and PI3K/Akt in Serum

**FIGURE 4:**
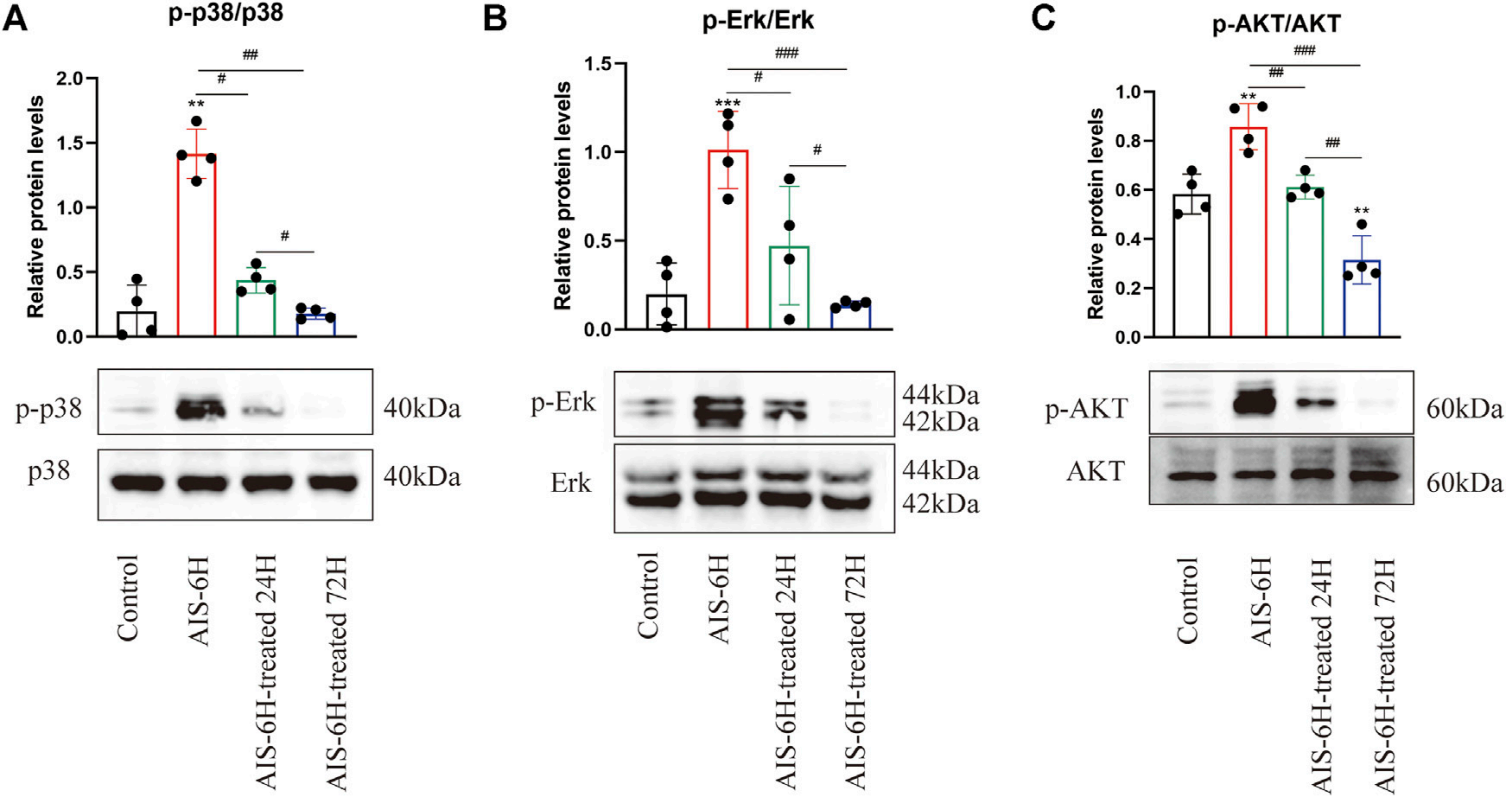
AIS promoted phosphorylation of p38 MAPK, Erk1/2, and PI3K/Akt in serum. **(A–C)** Phosphorylation levels of p38 MAPK, Erk1/2, and PI3K/Akt in serum were detected by Western blotting; *n* = 4. Data are presented as mean ± SD. Difference to control: **p < 0.01; ***p < 0.001; ^#^ Difference between groups: ^#^ *p* < 0.05; ^##^ *p* < 0.01; ^###^ *p* < 0.001; and one-way ANOVA with the Newman–Keuls multiple comparison test.

In order to identify the involvement of p38 MAPK, Erk1/2, and PI3K/Akt signaling pathways in the pathological process of AIS, Western blotting was performed, and it was observed that the phosphorylation levels of p38 MAPK, Erk1/2, and PI3K/Akt were significantly promoted in the AIS groups and decreased after rt-PA treatment (*p* < 0.05) (Figure 4), which indicated that p38 MAPK, Erk1/2, and PI3K/Akt pathways were involved in AIS progression.

### *miR-124-3p* Promoted Viability and Inhibited Apoptosis of Lipopolysaccharide-Induced BV2 Microglia

**FIGURE 5:**
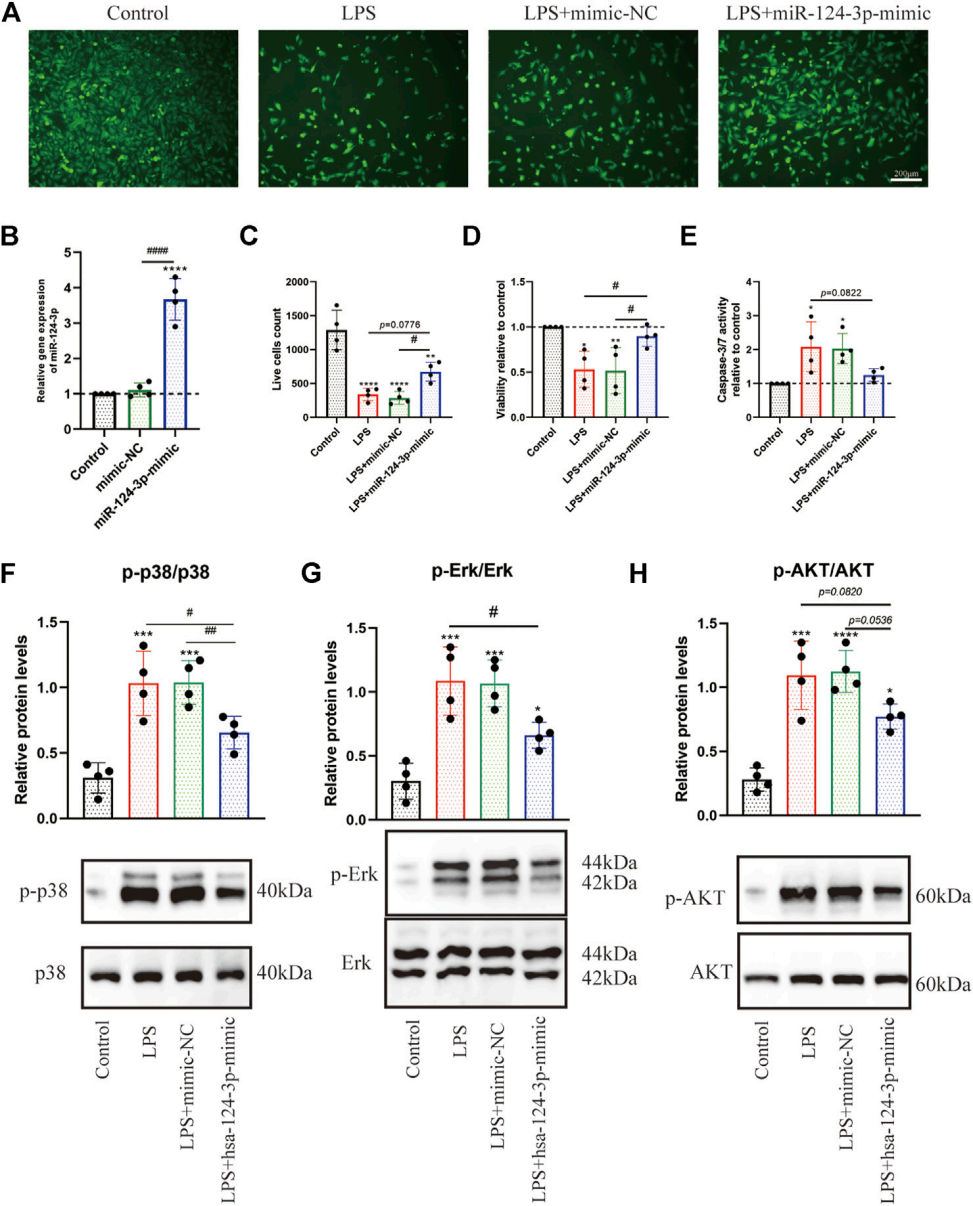
*hsa-miR-124-3p* mimic promoted viability, inhibited apoptosis, and reduced phosphorylation of Erk1/2, PI3K/Akt, and p38 MAPK in LPS-induced BV2 microglia. **(A,C)** Live cells were visualized by fluorescence microscopy after labeling cells with calcein. Living cells were labeled with calcein (green fluorescence); *n* = 4, scale bar = 200 μm. **(B)** Relative expression of transfected *hsa-miR-124-3p* mimic and mimic-NC measured by qRT-PCR in BV2 microglia, *n* = 4. (**D,E)** Viability and apoptosis analysis of LPS-induced BV2 microglia or/and transfected with *hsa-miR-124-3p* mimic. **(F–H)** Representative Western blotting images and quantification of the phosphorylation level of Erk1/2, P13K/Akt, and p38 MAPK after the densitometric analysis. All values represent mean ± SD. * compared with control, *p < 0.05; ***p < 0.001; ****p < 0.0001; ^#^ Difference between groups, ^*#*^ *p* < 0.05; ^*##*^ *p* < 0.01; ^*###*^ *p* < 0.001; one-way ANOVA with the Newman–Keuls multiple comparison test; *n* = 4.

To determine whether *miR-124-3p* regulates viability and apoptosis of LPS-induced BV2 microglia, cells were transfected with mimic-NC (NC = negative control) and *hsa-miR-124-3p-mimic* for 24 h. As shown in Figure 5A, the expression level of *hsa-miR-124-3p* was significantly (*p* < 0.05) upregulated by *hsa-miR-124-3p-mimic*. *hsa-miR-126-3p-mimic* significantly (*p* < 0.05) promoted viability and live cell count, and inhibited the caspase 3/7 activity of LPS-induced BV2 microglia (Figures 5B–E). *miR-124-3p* also exhibited a visible (*p* < 0.05) dose-dependent effect on viability and apoptosis of LPS-induced BV2 microglia (Supplementary Figure S1). These results suggested that *miR-124-3p* reversed the LPS-induced inflammatory effect in BV2 microglia.

### *miR-124-3p* Reduced the Phosphorylation of Erk1/2, PI3K/Akt, and p38MAPK, and Promoted Migration in Lipopolysaccharide-Induced BV2 Microglia

**FIGURE 6:**
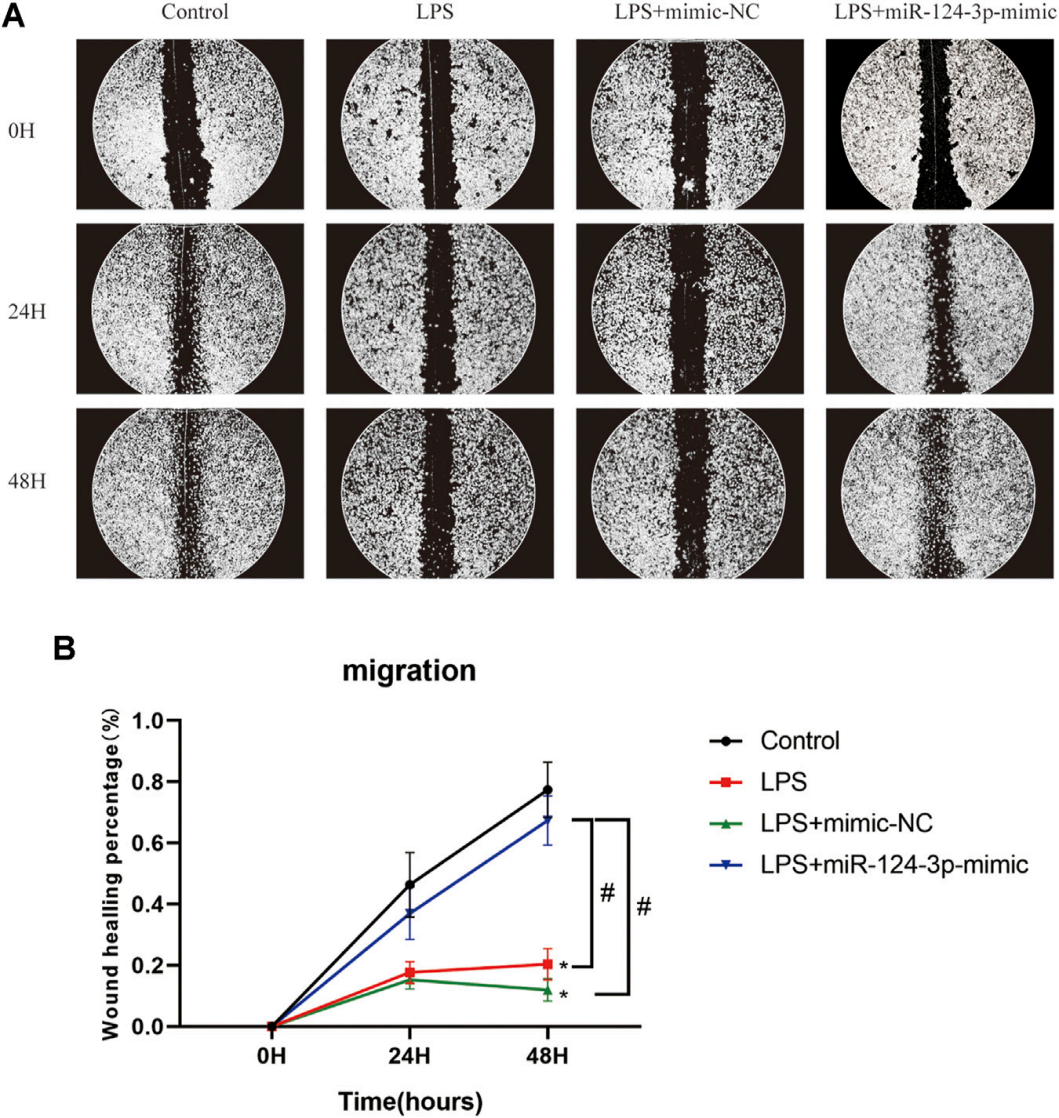
*miR-124-3p* promoted migration in LPS-induced BV2 microglia. **(A,B)** A scratch (wound healing) assay was used to determine the effect of *hsa-miR-124-3p* on the migration of LPS-induced BV2 microglia. The images of gaps were taken at 0, 24, and 48 h after treatment. The wound healing rate was used to calculate the migration ability of each group; *n* = 3. All values were represented as mean ± SD. Difference to control: *p < 0.05; ^#^ Difference between groups: ^#^ *p* < 0.05; one-way ANOVA with the Newman–Keuls multiple comparison test.

To evaluate the role of *miR-124-3p* in regulating pro-inflammatory signaling pathways activated by AIS, we examined the effect of *miR-124-3p* in LPS-induced BV2 microglia using Western blotting. The quantitative assessment showed that the phosphorylation of Erk1/2, PI3K/Akt, and p38 MAPK was promoted by LPS and significantly inhibited (*p* < 0.05) by *hsa-miR-124-3p-mimic* (Figures 5F–H). A scratch (wound healing) assay was performed to evaluate the effect of *miR-124-3p* on the migration of LPS-induced BV2 microglia. We observed a high wound healing (migration) rate in the control group and the *hsa-miR-124-3p-mimic* group compared to that in the LPS-treated group (*p* < 0.05) (Figures 6A,B). These findings indicated that *miR-124-3p* has therapeutic effects on LPS-induced BV2 microglia.

### *miR-124-3p* Inhibited the Expression of *GRB2* and *AKT3* in BV2 Microglia Cells

**FIGURE 7:**
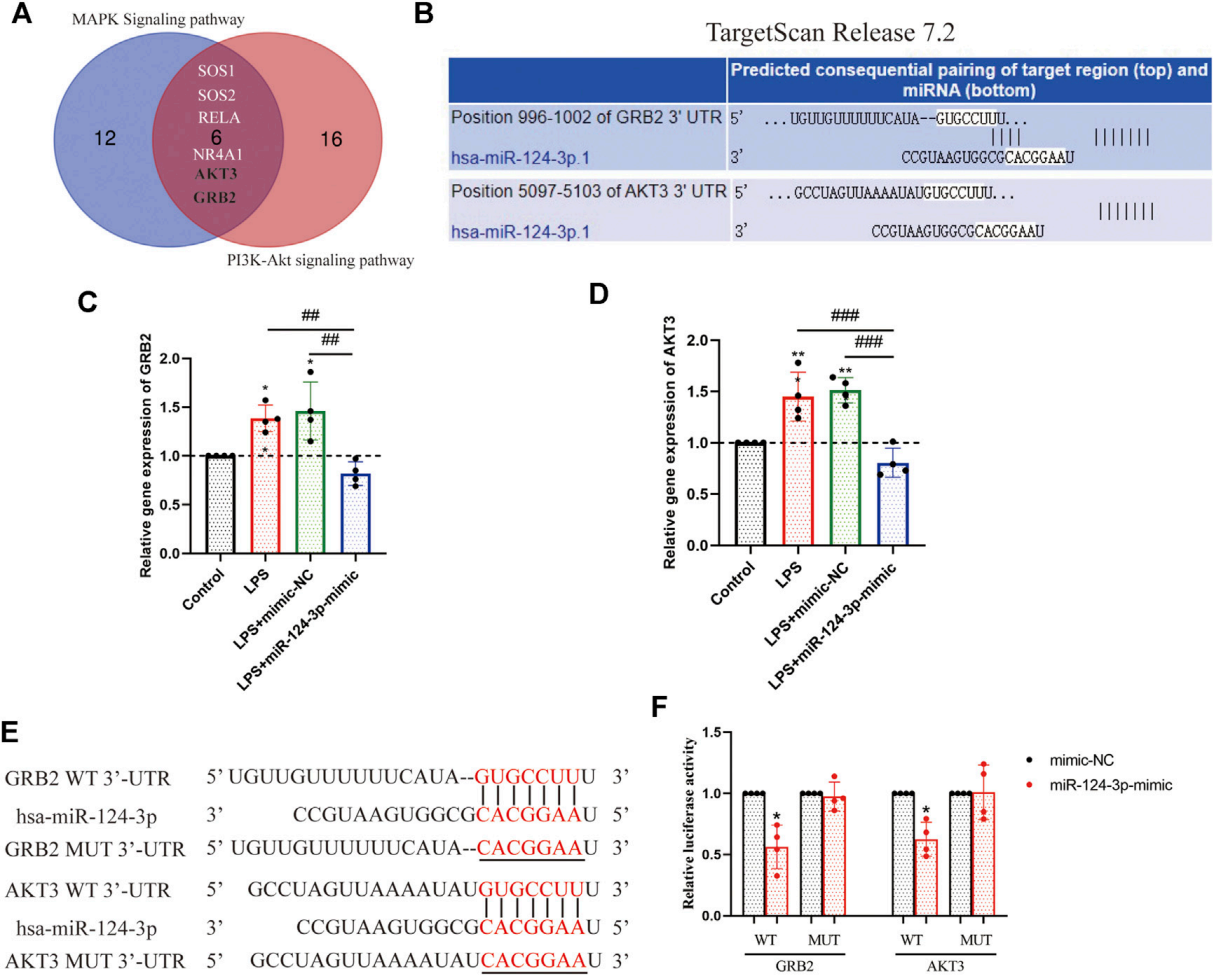
Identification of *GRB2* and *AKT3* as a direct target of *miR-124-3p* in BV2 microglia cells. **(A)** Venn diagram displayed the overlapping of the target genes of *miR-124-3p*, involved in MAPK signaling pathway and PI3K/AKT signaling pathway, as predicted in 2.3 and 2.4 of methods. **(B)** Putative *miR-124-3p* binding sequences in the 3′-UTR of *GRB2* and *AKT3* mRNA were predicted using the TargetScan database. **(C,D)** Gene expression analysis of *GRB2* and *AKT3* in BV2 microglia after LPS-induced or/and transfected with *miR-124-3p*-mimic. **(E)** The *miR-124-3p* binding site in the 3′UTR of *GRB2* and *AKT3* and the 3′UTR mutant sequences. **(F)** Qualification of luciferase activity for targeting the correlation between *miR-124-3p* and *GRB2/AKT3.* All values represent mean ± SD. * compared to control, *p < 0.05; **p < 0.01; ^#^ Difference between groups, ^##^ *p* < 0.01; ^###^ *p* < 0.001; one-way ANOVA with the Newman–Keuls multiple comparison test; *n* = 4.

To explore the mechanism of *miR-124-3p* in regulating pro-inflammatory signaling pathways, the luciferase reporter gene assay was performed to detect the correlation between key genes involved in pro-inflammatory signaling pathways and *miR-124-3p.* A total of six target genes of *miR-124-3p* screened out were involved in the MAPK signaling pathway and the PI3K/AKT signaling pathway; *GRB2* and *AKT3* were identified (Figure 7A). The potential binding sites of *GRB2* and *AKT3* 3′-untranslated region (UTR) are listed in Figure 7B. The expression of *GRB2* and *AKT3* genes was determined after 24 h of transfection with mimic-NC or hsa-miR-124-3p-mimic in LPS-induced BV2 microglia. The results showed that the expression of *GRB2* and *AKT3* genes was significantly (*p* < 0.01) inhibited by *miR-124-3p* (Figures 7C,D). The luciferase reporter assay suggested that (Figures 7E,F) the luciferase activity of the WT-*GRB2*-3′-UTR and WT-*AKT3*-3′-UTR in BV2 microglia cells was downregulated by *miR-124-3p*-mimic (*p* < 0.05). These results indicated that *miR-124-3p* directly targeted *GRB2* and *AKT3 via* binding the 3′-UTR in BV2 microglia.

## Discussion

Previous studies demonstrated that circulating miRNAs are altered in plasma/serum of AIS patients, serving as molecular markers for diagnosing and predicting the outcome (Jickling et al., 2010; Huang et al., 2016). However, the sensitivity and specificity of miRNAs for AIS diagnosis in plasma/serum were not satisfactory, especially in the early stage (Leung et al., 2014). In the present study, we observed that serum EV-derived *miR-124-3p* exhibited high sensitivity and accuracy in the diagnosis and prediction of AIS. In addition, the overexpression of *miR-124-3p* attenuates the inflammation in LPS-induced BV2 cells *via* regulating the pro-inflammatory signaling pathways.

**FIGURE 8:**
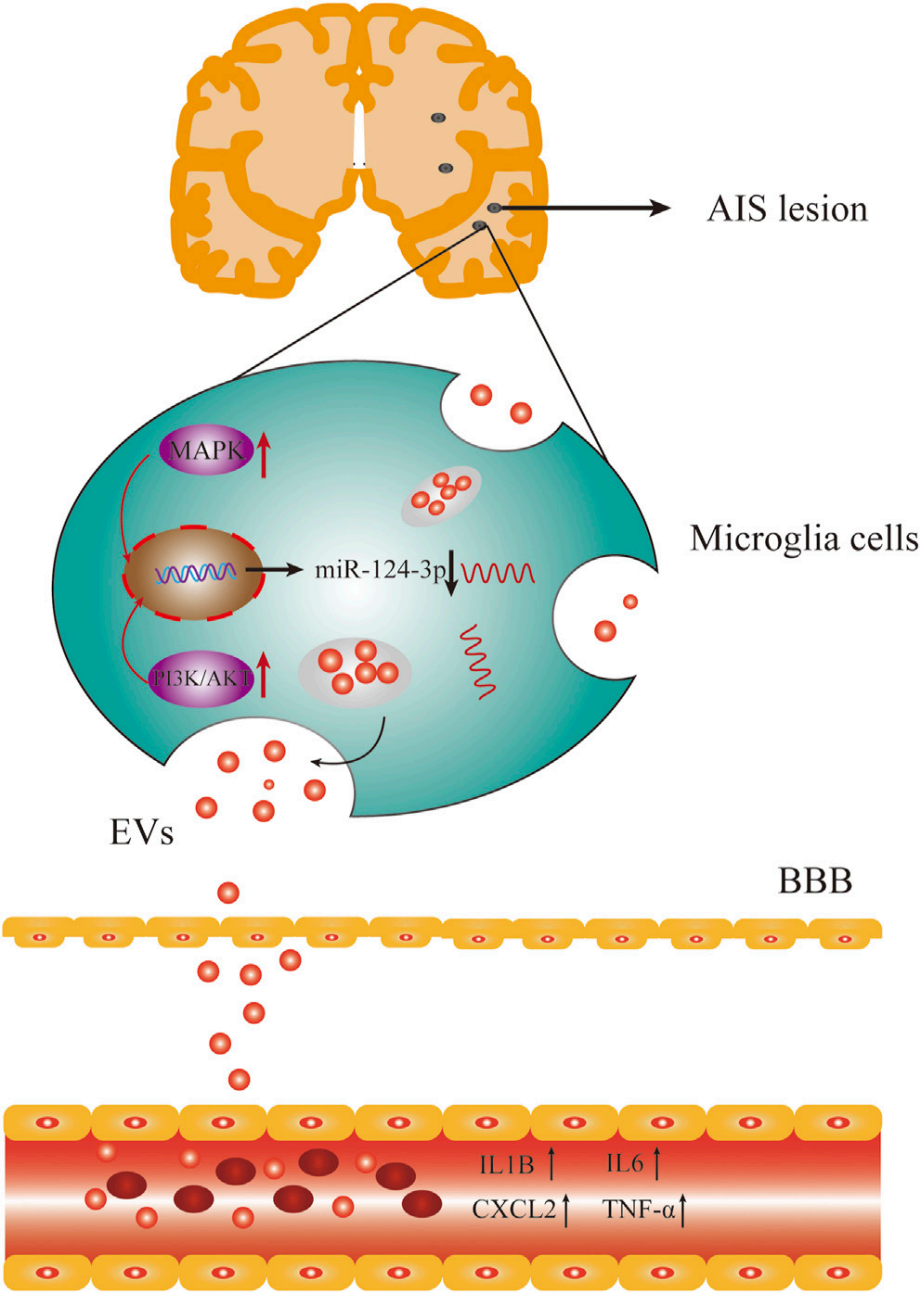
AIS activated the pro-inflammatory signaling pathways and inhibited the expression of *miR-124-3p*, which also upregulated the expression of pro-inflammatory factors in serum.

Accumulating evidence suggested that inflammation and immune responses play critical roles in the early stage of AIS and have been associated with the outcome of AIS (Wang, 2005; Chamorro and Hallenbeck, 2006). Previous studies demonstrated that pro-inflammatory cytokines increased during AIS, such as IL-6, CX3CL1, IL-1β, and TNF-α (Xiang et al., 2016; Pöyhönen et al., 2019). In this study, we found that the expression of IL1β, IL6, TNF-α, and CXCL2 was increased during the early stage of AIS (Figure 8) but decreased after treatment. We also observed that pro-inflammatory signaling pathways, such as p38 MAPK, Erk1/2, and PI3K/Akt, were activated during AIS, which was supported by previous studies (Liu et al., 2009; Lok et al., 2015; Fann et al., 2018; Zhang et al., 2021).

Reportedly, many miRNAs are differentially expressed in plasma/serum of AIS. Wang et al. (2014) found that *hsa-miR-106b-5p* and *hsa-miR-4306* are significantly increased in AIS patients, whereas *hsa-miR-320e* and *hsa-miR-320d* are significantly decreased in control subjects, suggesting that miRNAs in plasma might serve as biomarkers for the early diagnosis of acute stroke in humans. *miR-124-3p* is mainly expressed in the brain (Cheng et al., 2009). A previous study demonstrated that the expression of *miR-124-3p* was reduced substantially in focal cerebral ischemia *in vivo* (Liu et al., 2011). Liu et al. (2015) observed that the serum level of *miR-124* was significantly decreased within 24 h after stroke onset and negatively correlated with infarct size. In contrast, another study found that *miR-124* was significantly upregulated in AIS patients in the acute phase (Ji et al., 2016). In the present study, we found that EV *miR-124-3p* significantly decreased from 2 h of AIS onset and continued to decrease at 6 h of AIS onset. Notably, the expression of EV *miR-124-3p* is negatively correlated with *IL1β*, *IL6*, *TNF-α*, *CXCL2I*, and the NIHSS. Furthermore, EV *miR-124-3p* showed high sensitivity and specificity for the diagnosis of AIS compared to serum *miR-124-3p*.

*miR-124* has been identified as a negative regulator of inflammation after stroke (Hamzei Taj et al., 2016a; Ning et al., 2017). In the present study, we investigated the anti-inflammatory effect of *miR-124-3p* on LPS-induced BV2 microglia. The current results showed that *miR-124-3p* promoted migration and viability of LPS-induced BV2 microglia, and inhibited apoptosis and phosphorylation of pro-inflammatory signaling pathways (p38 MAPK, Erk1/2, and PI3K/Akt) activated by LPS. These results were consistent with those reported previously (Sun et al., 2013; Hamzei Taj et al., 2016b). In addition, EV miRNA was negatively correlated with pro-inflammatory cytokines (*CXCL2*, *IL6*, *IL1β*, and *TNF-α*). In order to reveal the mechanism underlying the role of *miR-124-3p* in regulating pro-inflammatory signaling pathways, a luciferase reporter gene assay was employed to detect the correlation between *miR-124-3p* and key genes involved in MAPK and PI3K/Akt signaling pathways. The results showed that *miR-124-3p* directly targeted *GRB2* and *AKT3* in BV2 microglia. This could be the mechanism of *miR-124-3p* inhibiting the activity of pro-inflammatory signaling pathways.

In conclusion, the current study demonstrated that serum EV-derived *miR-124-3p* exhibited high sensitivity and accuracy in the diagnosis and prediction of AIS. In addition, the overexpression of *miR-124-3p* attenuated inflammation in LPS-induced BV2 cells *via* regulating p38 MAPK, Erk1/2, and PI3K/Akt signaling pathways. These data indicated that serum EV-derived *miR-124-3p* can be used as a diagnostic and predictive marker for early-stage AIS.

## Data Availability

Publicly available datasets were analyzed in this study. These data can be found here. The raw datasets (GSE95204) used in this study were downloaded from the Gene Expression Omnibus (GEO) (http://www.ncbi.nlm.nih.gov/geo/).
