# Supplementary material for: Serum Extracellular Vesicle–Derived miR-124-3p as a Diagnostic and Predictive Marker for Early-Stage Acute Ischemic Stroke
Source: Front Mol Biosci. 2021 Jul 1;8:685088. doi: 10.3389/fmolb.2021.685088 (PMC8280338; doi:10.3389/fmolb.2021.685088)
Supplement: Supplementary file 1 [file Table1.DOCX]

#1.Analysis of differential gene expression and heatmap

logFoldChange=1

P=0.05

library(limma)

setwd("")

rt=read.table("genesymbolMatrix.txt",sep="\t",header=T,check.names=F)

rt=as.matrix(rt)

rownames(rt)=rt[,1]

exp=rt[,2:ncol(rt)]

dimnames=list(rownames(exp),colnames(exp))

rt=matrix(as.numeric(as.matrix(exp)),nrow=nrow(exp),dimnames=dimnames)

#differential

modType=c(rep("Control",3),rep("AIS",6))

design <- model.matrix(~0+factor(modType))

colnames(design) <- c("Control","AIS")

fit <- lmFit(rt,design)

cont.matrix<-makeContrasts(AIS-Control,levels=design)

fit2 <- contrasts.fit(fit, cont.matrix)

fit2 <- eBayes(fit2)

allDiff=topTable(fit2,adjust='fdr',number=200000)

write.table(allDiff,file="limmaTab.xls",sep="\t",quote=F,row.names=T)

#write table

diffSig <- allDiff[with(allDiff, (abs(logFC)>logFoldChange & P.Value < P )), ]

write.table(diffSig,file="diff.xls",sep="\t",quote=F,row.names=T)

diffUp <- allDiff[with(allDiff, (logFC>logFoldChange & P.Value < P )), ]

write.table(diffUp,file="up.xls",sep="\t",quote=F,row.names=T)

diffDown <- allDiff[with(allDiff, (logFC<(-logFoldChange) & P.Value < P )), ]

write.table(diffDown,file="down.xls",sep="\t",quote=F,row.names=T)

#write expression level of diff gene

diffSig=read.table("diff.xls",sep="\t",header=T,check.names=F)

hmExp=rt[as.vector(diffSig[,1]),]

diffExp=rbind(id=colnames(hmExp),hmExp)

write.table(diffExp,file="diffExp.txt",sep="\t",quote=F,col.names=F)

library(pheatmap)

hmExp=rt[rownames(diffSig),]

Type=c(rep("Control",3),rep("AIS",3),rep("AIS-treated",3))

names(Type)=colnames(rt)

Type=as.data.frame(Type)

pdf(file="mirnaHeatmap.pdf",height=12,width=15)

pheatmap(hmExp,

annotation=Type,

color = colorRampPalette(c("green", "black", "red"))(50),

cluster_cols =F,

#show_colnames = F,

scale="row",

fontsize = 12,

fontsize_row=3,

fontsize_col=10)

dev.off()

#2. GO and KEGG pathway enrichment

#KEGG pathway enrichment

library("clusterProfiler")

rt=read.table("id.txt",sep="\t",header=T,check.names=F)

rt=rt[is.na(rt[,"entrezID"])==F,]

NormFit=rt$NormFit

gene=rt$entrezID

names(NormFit)=gene

kk <- enrichKEGG(gene = gene, organism = "hsa", pvalueCutoff =0.05, qvalueCutoff =0.05)

write.table(kk,file="KEGG.txt",sep="\t",quote=F,row.names = F)

tiff(file="barplot.tiff",width = 35,height = 22,units ="cm",compression="lzw",bg="white",res=300)

barplot(kk, drop = TRUE, showCategory = 20)

dev.off()

#GO enrichment

library("clusterProfiler")

library("org.Hs.eg.db")

rt=read.table("id.txt",sep="\t",header=T,check.names=F)

rt=rt[is.na(rt[,"entrezID"])==F,]

NormFit=rt$NormFit

gene=rt$entrezID

names(NormFit)=gene

kk <- enrichGO(gene = gene,OrgDb = org.Hs.eg.db, pvalueCutoff =0.05, qvalueCutoff = 0.05)

write.table(kk,file="GO.txt",sep="\t",quote=F,row.names = F)

tiff(file="barplot.tiff",width =35,height = 20,units ="cm",compression="lzw",bg="white",res=300)

barplot(kk, drop = TRUE, showCategory =20)

dev.off()

#3.multiVarROC

library(pROC)

inputFile="input.txt"

outFile="ROC.pdf"

setwd("")

rt=read.table(inputFile,header=T,sep="\t",check.names=F,row.names=1)

y=colnames(rt)[1]

bioCol=c("red","blue","green","yellow")

if(ncol(rt)>4){

bioCol=rainbow(ncol(rt))}

pdf(file=outFile,width=5,height=5)

roc1=roc(rt[,y], as.vector(rt[,2]))

aucText=c( paste0(colnames(rt)[2],", AUC=",sprintf("%0.3f",auc(roc1))) )

plot(roc1, col=bioCol[1])

for(i in 3:ncol(rt)){

roc1=roc(rt[,y], as.vector(rt[,i]))

lines(roc1, col=bioCol[i-1])

aucText=c(aucText, paste0(colnames(rt)[i],", AUC=",sprintf("%0.3f",auc(roc1))) )

}

legend("bottomright", aucText,lwd=2,bty="n",col=bioCol[1:(ncol(rt)-1)])

dev.off()

#4.Correlation analysis

library(ggplot2)

library(ggpubr)

library(ggExtra)

inputFile="input.txt"

gene1="gene1name"

gene2="gene1name"

rt=read.table(inputFile,sep="\t",header=T,check.names=F,row.names=1)

x=as.numeric(rt[gene1,])

y=as.numeric(rt[gene2,])

df1=as.data.frame(cbind(x,y))

corT=cor.test(x,y,method="spearman")

cor=corT$estimate

pValue=corT$p.value

p1=ggplot(df1, aes(x, y)) +

xlab(gene1)+ylab(gene2)+

geom_point()+ geom_smooth(method="lm",formula = y ~ x) + theme_bw()+

stat_cor(method = 'pearson', aes(x =x, y =y))

p2=ggMarginal(p1, type = "density", xparams = list(fill = "orange"),yparams = list(fill = "blue"))

pdf(file="cor.density.pdf",width=5,height=5)

print(p2)

dev.off()
