# Supplementary figures and images for: Serum Extracellular Vesicle–Derived miR-124-3p as a Diagnostic and Predictive Marker for Early-Stage Acute Ischemic Stroke
Source: Front Mol Biosci. 2021 Jul 1;8:685088. doi: 10.3389/fmolb.2021.685088 (PMC8280338; doi:10.3389/fmolb.2021.685088)

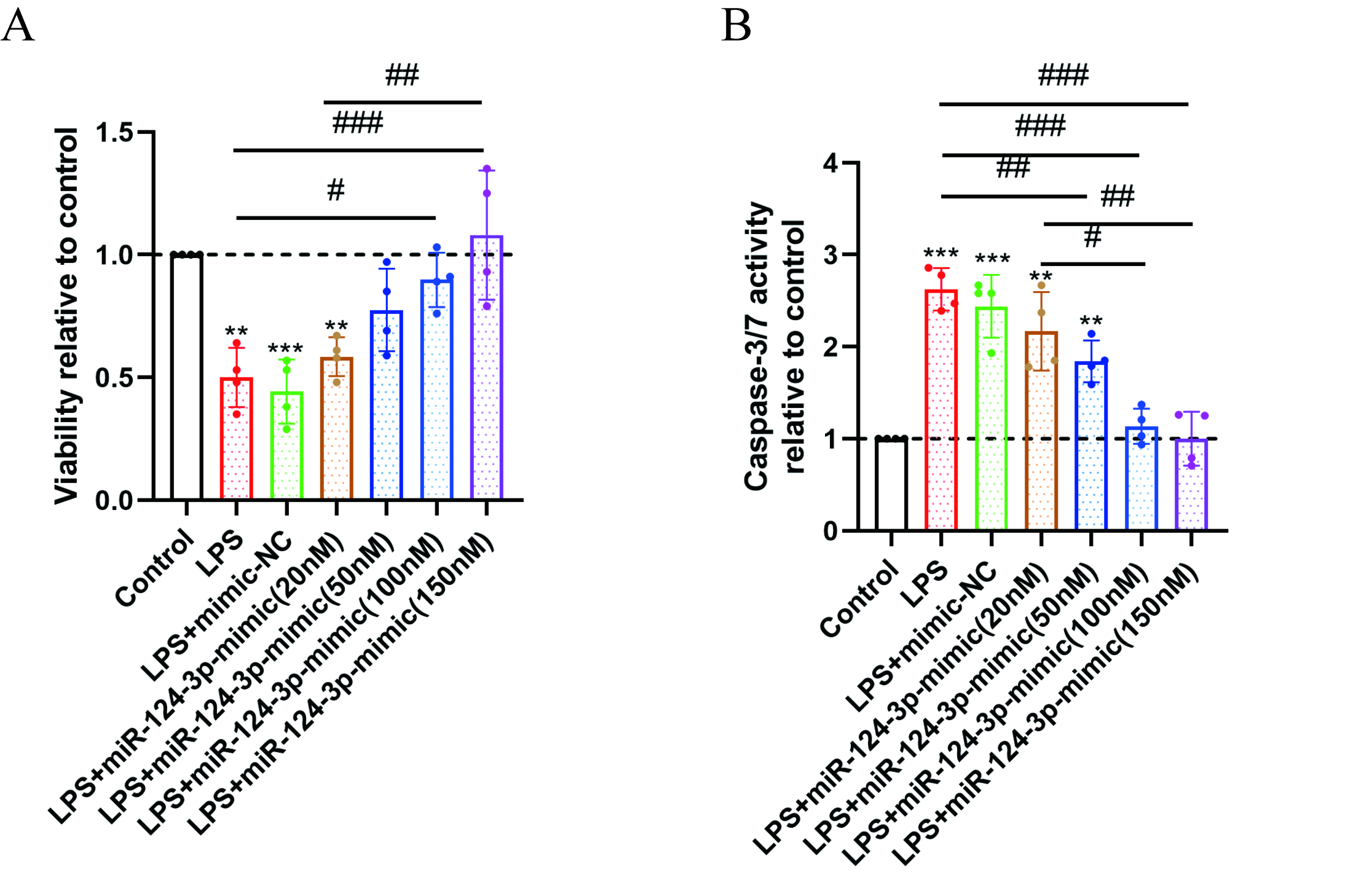

Supplement: Supplementary file 2 [file Image1.TIF]
